# Sensing Properties of NH_2_-MIL-101 Series for Specific Amino Acids via Turn-On Fluorescence

**DOI:** 10.3390/molecules26175336

**Published:** 2021-09-02

**Authors:** Jing Dong, Xiao-Yao Dao, Xiao-Yu Zhang, Xiu-Du Zhang, Wei-Yin Sun

**Affiliations:** 1 Coordination Chemistry Institute, State Key Laboratory of Coordination Chemistry, School of Chemistry and Chemical Engineering, Nanjing National Laboratory of Microstructures, Collaborative Innovation Center of Advanced Microstructures, Nanjing University, Nanjing 210023, China; jdong@njtech.edu.cn (J.D.); DG1724013@smail.nju.edu.cn (X.-Y.D.); DG20240143@smail.nju.edu.cn (X.-Y.Z.); 2 Scientific Research Department, Nanjing Tech University, Nanjing 211816, China; 3 College of Chemistry and Materials Science, Key Laboratory of Functional Molecular Solids Ministry of Education, Anhui Laboratory of Molecule-Based Materials, Anhui Key Laboratory of Functional Molecular Solids, Anhui Normal University, Wuhu 241002, China

**Keywords:** metal–organic frameworks, MIL-101, amino acids, fluorescence sensing, turn-on effect

## Abstract

Metal–organic frameworks (MOFs) have been demonstrated to be desired candidates for sensing definite species owing to their tunable composition, framework structure and functionality. In this work, the NH_2_-MIL-101 series was utilized for sensing specific amino acids. The results show that cysteine (Cys) can significantly enhance the fluorescence emission of NH_2_-MIL-101-Fe suspended in water, while NH_2_-MIL-101-Al exhibits the ability to sense lysine (Lys), arginine (Arg) and histidine (His) in aqueous media via turn-on fluorescence emission. Titration experiments ensure that NH_2_-MIL-101-Fe and NH_2_-MIL-101-Al can selectively and quantitatively detect these amino acids. The sensing mechanism was examined and discussed. The results of this study show that the metal centers in MOFs are crucial for sensing specific amino acids.

## 1. Introduction

Amino acids (AAs) are important organic compounds with amino and carboxyl groups and also the essential building units of proteins and enzymes [1]. They are indispensable nutrient compositions in living organisms that are crucially involved in almost all life activities. Abnormal physiological amino acid levels in organisms usually lead to various diseases or serious physiological dysfunctions such as cardiovascular diseases, neurological diseases, diabetes, hepatic failure, kidney malfunctioning, Alzheimer’s disease and schizophrenia [2,3,4]. Although there are only 20 basic natural AAs, the combinations of these AAs in different ways make up a tremendous amount of proteins with abundant functions and each amino acid plays an individual characteristic role. For example, cysteine (Cys) is the only one containing the sulfhydryl group among the natural amino acids that may contribute to regulating redox homeostasis and maintaining the spatial structure of proteins [5,6]. Whether an excess or deficiency of Cys could cause some heavy diseases such as abnormal hematopoiesis, neurotoxicity, Alzheimer’s disease, retarded growth, edema, muscle/fat loss, hair depigmentation, skin lesions, liver damage and so on [6,7,8]. Lysine (Lys) is a kind of important essential amino acid that cannot be manufactured by the body itself and, thus, must be taken in through daily diet. Lys plays crucial roles in varied biological processes and metabolism, such as the Krebs–Henseleit cycle, polyamine synthesis, carnitine production and so forth [9,10,11]. Meanwhile, the amount of Lys is considered a criterion to evaluate the nutritional level of food as well. Arginine (Arg) is the only one with a guanidine group and the most alkaline one among the natural amino acids. It makes a great contribution to regulating hormone levels and maintaining blood pressure and the immune system, and could also be used to treat various physiological diseases by enhancing vasodilation, such as cardiovascular diseases, erectile dysfunction, peripheral vascular diseases, vascular headaches, atherosclerosis and chest pain [12,13]. Moreover, Arg is commonly considered a biomarker of cystinuria (aka “sulfite oxidase deficiency”) and certain auxotrophic tumors in medical diagnosis [13,14]. As another essential natural amino acid, histidine (His) plays an indispensable role as neurotransmitters or neuromodulators in the central nervous system of mammals [15]. Previous studies have demonstrated that excessive His in the body would lead to poisoning symptoms, thrombotic disorders and mental diseases, including anxiety and schizophrenia, while its deficiency would also result in some damage to the nutritional status of patients suffering from chronic kidney diseases [16,17]. Therefore, the detection of specific amino acid is of great significance for the diagnosis, treatment and prognosis of disease, and thus the development of sensors toward amino acids has attracted a great deal of attention in recent years [18].

Metal–organic frameworks (MOFs) are a family of inorganic–organic hybrid materials assembled via the coordination between metal ions or metal clusters and organic bridging ligands. MOFs are of high diversity in structures and properties, typically showing advantages of high porosity, high specific surface area, openly accessible functional sites, tunable pore size and high chemical/thermal stability [19]. Due to these superior features, MOFs have been successfully used in different areas including heterogeneous catalysis [20], gas adsorption and separation [21,22], chemical sensing [23], drug delivery [24], bioimaging [25], etc. As for the luminescent MOFs, the guest species introduced may interact with the backbones of MOFs and result in the enhancement or suppression of luminescence, thereby enabling the application of luminescent MOFs in chemical sensing [26,27]. As luminescence-sensing platforms, MOFs have gained great success in the detection of a wide range of substances or parameters, including small molecules [28], biomolecules [29], explosives [30], cations/anions [31], gas molecules [32], humidity [33], temperature [34] and pH [35]. Compared to other luminescent sensing materials, the high porosity and large specific surface area make MOFs capable of encapsulating different guest species into their pores, which is beneficial to strengthening the interactions between the framework and guest species [36]. Furthermore, the modification of MOFs with various functionalized groups could help to generate specific sensing sites, such as open metal sites, Lewis acid/base sites and so on, toward guest molecules, which could improve the sensing performance of MOFs or even endow MOFs with the sensing capacity [37]. For example, our recent work has demonstrated that the amino-functionalized UiO-66 could be used as fluorescent sensor for Lys and Arg, while the parent UiO-66 without the amino group does not exhibit such a sensing capacity for amino acids [38]. 

In the past several decades, plenty of efforts have been devoted to the design and synthesis of MOFs, and thousands of novel MOFs with different structures have been reported, such as the major subclasses of UiO [39], ZIF [40], MIL [41], IRMOF [42] and PCN [43]. Among these classic MOFs, the MIL-101 series has been widely investigated due to the ultrahigh porosity, large specific surface area, easy modification, multi-functionalities, and particularly the diversity of metal centers [44]. Therefore, as a continuation of our previous work, we investigated the feasibility of amino-functionalized MIL-101-Fe and MIL-101-Al, namely NH_2_-MIL-101-Fe and NH_2_-MIL-101-Al, as fluorescent sensors toward specific amino acids in this work. The results of sensing experiments indicate that Cys could significantly enhance the fluorescence emission intensity of NH_2_-MIL-101-Fe suspended in water among the natural amino acids, while NH_2_-MIL-101-Al exhibits turn-on fluorescence emission response to Lys, Arg and His. Meanwhile, the fluorescence titration experiments suggest that all the enhancement efficiencies of Cys for NH_2_-MIL-101-Fe and Lys/Arg/His for NH_2_-MIL-101-Al are proportional to the concentration of the analyte within a certain range, which made it possible for the quantitative determination of these amino acids. Moreover, in the presence of interfering AAs, NH_2_-MIL-101-Fe/NH_2_-MIL-101-Al could still specifically detect the target analyte by giving a significantly enhanced fluorescence response, except that the presence of Lys, Asp (L-aspartic acid), His and Arg could obviously affect the sensing capacity of NH_2_-MIL-101-Fe toward Cys, indicating their reasonable anti-interference. Moreover, the sensing mechanism of NH_2_-MIL-101-Fe toward Cys and of NH_2_-MIL-101-Al toward Lys/Arg could be ascribed to the structural collapse, while the sensing capacity of NH_2_-MIL-101-Al toward His was concerned with the adsorption of His into the voids of NH_2_-MIL-101-Al.

## 2. Results and Discussion

The phase purities of the bulk as-synthesized powder samples of NH_2_-MIL-101-Fe and NH_2_-MIL-101-Al were ensured by measurements of PXRD (powder X-ray diffraction). As illustrated in Figure S1, the PXRD patterns of the as-synthesized NH_2_-MIL-101-Fe and NH_2_-MIL-101-Al are identical to the simulated one generated from the data of single-crystal X-ray diffraction analysis, demonstrating their phase purities. In addition, the fluorescence properties of NH_2_-MIL-101-Fe and NH_2_-MIL-101-Al in the solid state and in aqueous suspension were investigated at room temperature. As shown in Figure S2, it can be found that NH_2_-MIL-101-Fe and NH_2_-MIL-101-Al exhibit weak emission with maxima at 485 and 450 nm under the excitation of 350 nm in the solid state, respectively, while the fluorescence emissions of the suspensions of NH_2_-MIL-101-Fe and NH_2_-MIL-101-Al show some blue shift and enhancement with the maxima at 454 and 440 nm upon excitation at 356 and 340 nm, respectively.

**Figure 1:**
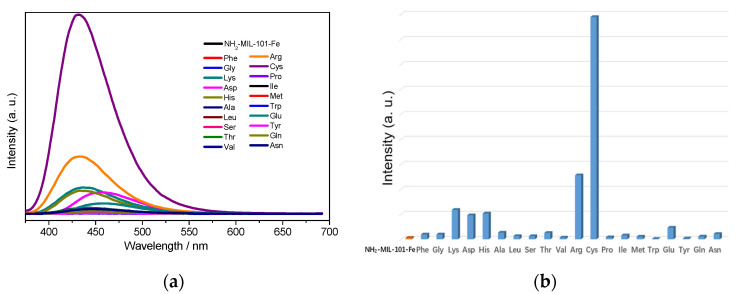

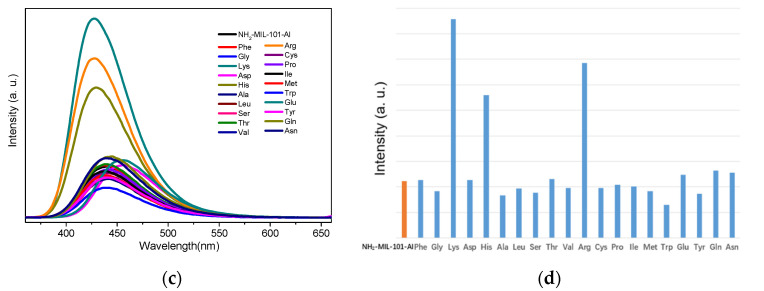
Emission spectra of NH_2_-MIL-101-Fe (**a**) and NH_2_-MIL-101-Al (**c**) in aqueous suspension without and with different AAs. Fluorescence peak intensities of the aqueous suspension of NH_2_-MIL-101-Fe (**b**) and NH_2_-MIL-101-Al (**d**) without and with different AAs. (The emission spectra were achieved with an optical attenuator due to the measuring range of fluorescence intensity).

Next, in order to explore the sensing capacity of NH_2_-MIL-101-Fe and NH_2_-MIL-101-Al toward amino acids, their fluorescence emission spectra in the suspension of 20 kinds of different natural amino acids including Ala (L-alanine), Arg (L-arginine), Asn (L-asparagine), Asp (L-aspartic acid), Cys (L-cysteine), Glu (L-glutamic acid), Gln (L-glutamine), Gly (glycine), His (L-histidine), Ile (L-isoleucine), Leu (L-leucine), Lys (L-lysine), Met (L-methionine), Phe (L-phenylalanine), Pro (L-proline), Ser (L-serine), Thr (L-threonine), Trp (L-tryptophan), Tyr (L-tyrosine) and Val (L-valine) with a concentration of 0.1 M were recorded in the range of 375–690 and 360–660 nm excited at 356 and 340 nm, respectively. As depicted in Figure 1, it was worth noting that the emission of NH_2_-MIL-101-Fe was tremendously enhanced by Cys (up to 158 times), while significant increments in the emission intensities in the suspension of Lys (about 3.9 times), Arg (about 3.1 times) and His (about 2.5 times) could be observed for NH_2_-MIL-101-Al.

**Figure 2:**
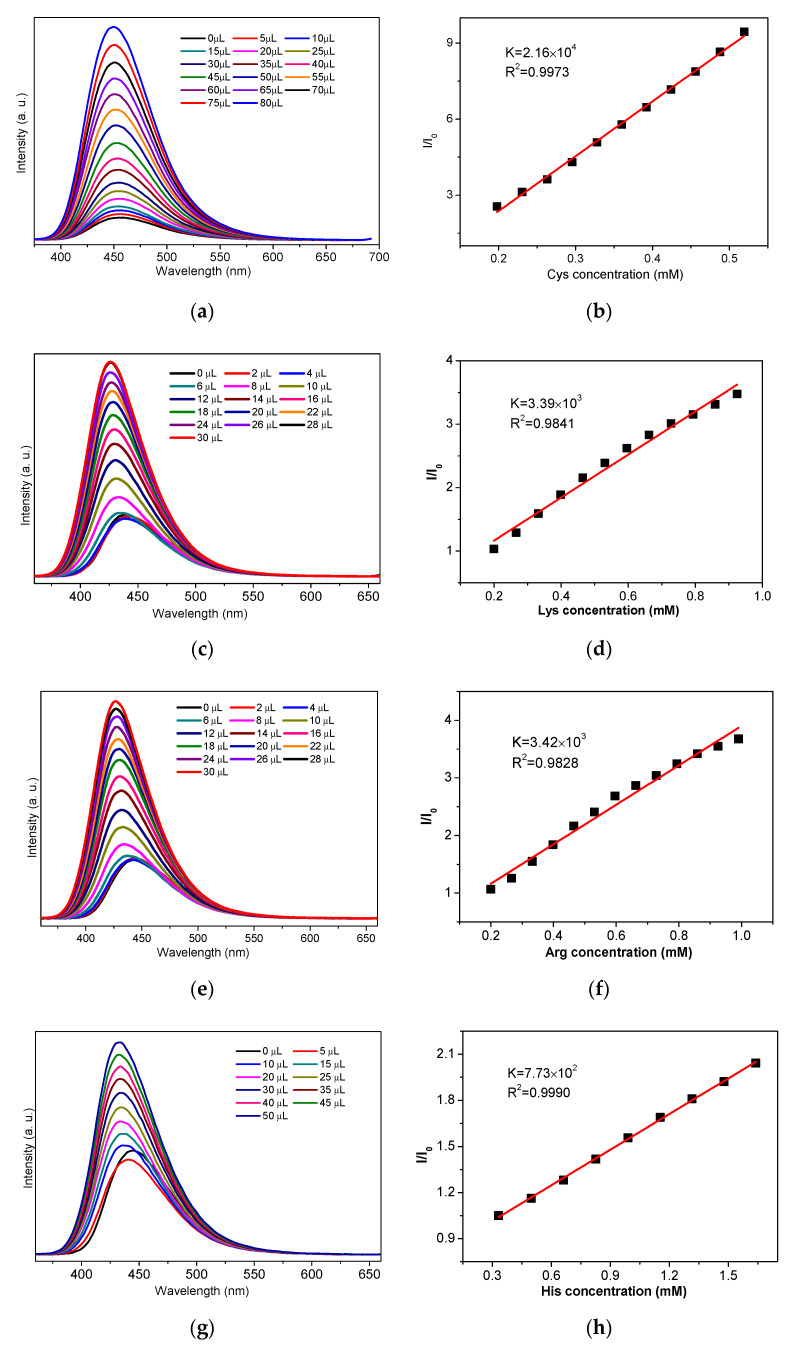
The changes in the emission intensity of the aqueous suspension of NH_2_-MIL-101-Fe upon the gradual addition of Cys (**a**) excited at 356 nm. The changes in the emission intensity of the aqueous ethanol suspension of NH_2_-MIL-101-Al upon the gradual addition of Lys (**c**)/Arg (**e**)/His (**g**) excited at 340 nm. The *I*/*I*_0_ versus the concentration plots of Cys (**b**)/Lys (**d**)/Arg (**f**)/His (**h**). (The emission spectra of NH_2_-MIL-101-Al were obtained by using an optical attenuator owing to the measuring range of fluorescence intensity).

The above fluorescence turn-on effect of Cys for NH_2_-MIL-Fe and Lys/Arg/His for NH_2_-MIL-Al indicates the possibility for the detection of specific amino acids. To further investigate the sensing sensitivity of NH_2_-MIL-101-Fe toward Cys and NH_2_-MIL-101-Al for Lys/Arg/His, titration experiments with gradual addition of Cys in H_2_O (0.02 M) to the aqueous suspension of NH_2_-MIL-101-Fe (0.5 mg/mL, 3 mL) and the solution of Lys/Arg/His in water (0.1 M) to the suspension of NH_2_-MIL-101-Al (0.5 mg/mL, 3 mL) were performed, respectively. As shown in Figure 2a,c,e,g, it can be observed that all the fluorescence emission intensities of the suspension of NH_2_-MIL-101-Fe and NH_2_-MIL-101-Al increased continuously along with the gradual addition of Cys and Lys/Arg/His. It is worth noting that in the titration of His (Figure 2g), the emission decreases first since the 0 µL data (black) is higher than the 5 µL data (red). This decreased and shifted emission is considered to originate from the His-encapsulated NH_2_-MIL-101-Al (vide post) with weak intensity owing to the low concentration of His, and after that, the emission increases upon further addition of His. Furthermore, the emission enhancement ratios *I*/*I*_0_ (*I*_0_ and *I* are the fluorescence intensities of the suspension without and with the presence of the aqueous solution of the amino acid) of NH_2_-MIL-101-Fe and NH_2_-MIL-101-Al exhibited a linear relationship with the corresponding concentration of Cys and Lys/Arg/His within the concentration ranges of 0.2–0.5, 0.2–0.9, 0.2–1.0 and 0.3–1.6 mM, and the correlation coefficients (R^2^) are 0.9973 (Figure 2b), 0.9841 (Figure 2d), 0.9828 (Figure 2f) and 0.9990 (Figure 2h) for Cys, Lys, Arg and His, respectively. Then, we employed the equation *I*/*I*_0_ = K[A] + 1 to match the linear relationship, in which K is the slope and [A] is the concentration of the amino acids added to the suspension. According to this formula, the K value of the linear curves could be estimated to be 2.16 × 10^4^ M^−1^ for Cys, 3.39 × 10^3^ M^−1^ for Lys, 3.42 × 10^3^ M^−1^ for Arg and 7.73 × 10^2^ M^−1^ for His. Meanwhile, the limit of detection (LOD) could also be determined, using the standard equation 3δ/K, to be 10.1, 45.5, 45.1 and 199 μM toward Cys, Lys, Arg and His for NH_2_-MIL-101-Fe and NH_2_-MIL-101-Al, respectively, in which δ represents the standard deviation for repeating experiments of the suspensions. 

**Figure 3:**
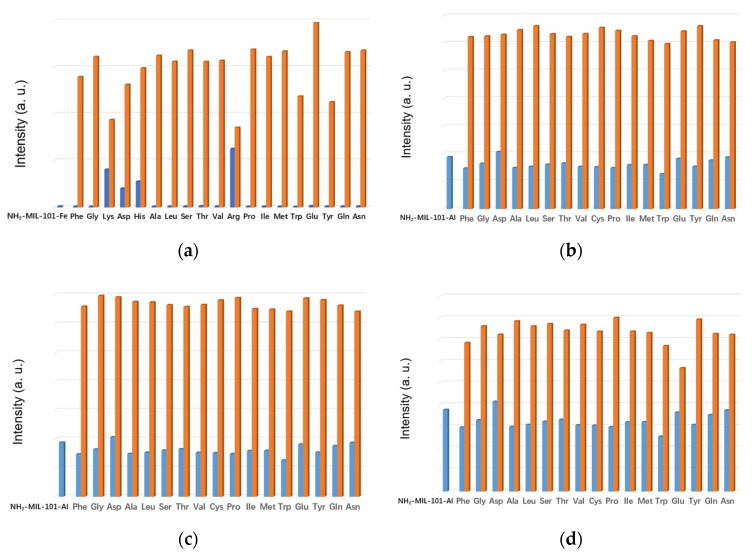
The emission intensities of the suspension of NH_2_-MIL-101-Fe with addition of competing AAs followed by addition of Cys (**a**) excited at 356 nm. The emission intensities of the suspension of NH_2_-MIL-101-Al with addition of competing AAs followed by addition of Lys (**b**)/Arg (**c**)/His (**d**) excited at 340 nm. (The emission spectra were obtained by using an optical attenuator owing to the measuring range of the emission intensity).

As we know, the anti-interfering capability is one of the most important evaluation criteria for sensing materials. Therefore, we carried out competing experiments through the first addition of other AAs in H_2_O followed by addition of Cys and Lys/Arg/His to the corresponding suspension of NH_2_-MIL-101-Fe and NH_2_-MIL-101-Al to verify their anti-interference. For NH_2_-MIL-101-Fe, the addition of all the other interfering amino acids had no significant effect on the sensing capability toward Cys except Lys, Asp, His and Arg (Figure 3a). It can be observed that the addition of Lys, Asp, His or Arg could also enhance the emission intensity of NH_2_-MIL-101-Fe, which only resulted in a limited increment in the emission intensity upon the successive addition of Cys. Meanwhile, it is clearly indicated in Figure 3b–d that the presence of other amino acids had no significant influence on the enhancing effect of Lys/Arg/His for the fluorescence emission intensities of NH_2_-MIL-101-Al. These phenomena implied that NH_2_-MIL-101-Fe exhibited reasonable anti-interference except Lys, Asp, His and Arg while NH_2_-MIL-101-Al could still be used for sensing Lys/Arg/His in the existence of other amino acids. 

**Figure 4:**
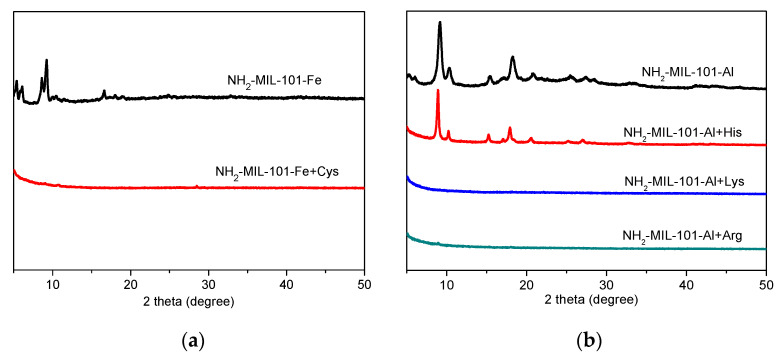
PXRD data of NH_2_-MIL-101-Fe (**a**) and NH_2_-MIL-101-Al (**b**) before and after soaking in the aqueous solution of Cys and Lys/Arg/His.

In addition, we also made efforts to investigate the sensing mechanism of NH_2_-MIL-101-Fe and NH_2_-MIL-101-Al toward specific amino acids. Generally, the primary concern of the sensing mechanism of MOFs is the structural stability of the sensing materials. Therefore, we employed PXRD to examine the stability of NH_2_-MIL-101-Fe and NH_2_-MIL-101-Al in the solution of amino acids. As shown in Figure 4, only the PXRD patterns of NH_2_-MIL-101-Al in the presence of His matched well with those of the as-synthesized sample, suggesting the structural stability of NH_2_-MIL-101-Al in the aqueous solution of His, while the PXRD peaks of NH_2_-MIL-101-Fe in the aqueous solution of Cys and NH_2_-MIL-101-Al in the aqueous solution of Lys/Arg all disappeared, indicating the collapse of the structures of NH_2_-MIL-101-Fe and NH_2_-MIL-101-Al. Therefore, it could be concluded preliminarily that the sensing capability of NH_2_-MIL-101-Fe toward Cys and of NH_2_-MIL-101-Al toward Lys and Arg could be attributed to the structural collapse and such mechanisms can be excluded for NH_2_-MIL-101-Al toward His. To further investigate the intrinsic reason, we speculate that it is the encapsulation of His into the pores of NH_2_-MIL-101-Al that causes the fluorescence emission intensity enhancement based on the investigation of our previous work [38]. Thus, we employed ^1^H NMR and FT-IR measurements to verify this speculation. After digesting in NaOH/ D_2_O, we measured the ^1^H NMR spectra of the untreated and His-treated NH_2_-MIL-101-Al in D_2_O, and the results are depicted in Figure S3. Compared to the ^1^H NMR spectra of NH_2_-MIL-101-Al, it could be found that some new peaks belonging to His appeared in that of His-treated NH_2_-MIL-101-Al (Figure S3a), suggesting the adsorption of His into the pores of NH_2_-MIL-101-Al. In addition, it could be observed that a new C-C stretching vibration band belonging to His around 1142 cm^−1^ appeared and the O-H stretching bands belonging to solvent water molecules were greatly weakened in the FT-IR spectra of His-treated NH_2_-MIL-101-Al (Figure S3b), suggesting that the solvent molecules were exchanged by His. Such a mechanism of collapse of the framework or encapsulation of the analyte into the pore of the framework makes the linear sensing response in the mM range (Figure 2), rather than µM. It means that the amount of analyte in the mM range is essential to destroy the framework or to enter the pore of the framework, resulting in enhancement of fluorescence emission, while the fluorescence sensing with electron transfer and/or energy transfer mechanism gives high sensitivity in the µM range [45]. Furthermore, alternative chemical spectroscopy approaches such as surface-enhanced Raman scattering (SERS) spectroscopy can provide very high sensitivity with a range as low as nM or even pM and aM concentrations [46,47]. In short, the sensing sensitivity depends on mechanism as well as chemical spectroscopy. 

Finally, another phenomenon also attracted our interest: the fluorescence emission enhancement ratio of Cys for NH_2_-MIL-101-Fe was so high. In consideration of the oxidizing capacity of the metal centers of NH_2_-MIL-101-Fe and the reducing ability of Cys together with the structural collapse of NH_2_-MIL-101-Fe in the solution of Cys, we speculated that it may be attributed to the redox reaction between Cys and the metal centers of Fe^3+^ in NH_2_-MIL-101-Fe. To verify this, ^1^H NMR and high-resolution mass spectrometry (HRMS) measurements were carried out. As shown in Figure S4, it could be found that the ^1^H NMR peaks of Cys-treated NH_2_-MIL-101-Fe are different from those of Cys. Furthermore, the HRMS experiment also demonstrated the existence of the cystine ([M + H]^+^, *m*/*z* = 241.0307, calcd. 241.0311), which is regarded as the oxidation product of Cys. The results show that the redox reaction occurred when Cys meets NH_2_-MIL-101-Fe, leading to the collapse of the framework. While in the case of Lys and Arg, the NH_2_-MIL-101-Al framework may be destroyed by the functional groups in the side chain of Lys and Arg, which can interact with and attack the framework. Furthermore, the destroying NH_2_-MIL-101-Fe and NH_2_-MIL-101-Al frameworks with the presence of Cys/Lys/Arg release the amino-benzene dicarboxylate ligand (NH_2_-BDC), and thus enhance the fluorescence emission because the emission intensity of NH_2_-BDC is stronger than those of NH_2_-MIL-101-Fe and NH_2_-MIL-101-Al in aqueous suspension (Figure S5). On the other hand, reversibility and recyclability are also not available for NH_2_-MIL-101-Al to detect His, which may be reasoned by strong interactions between His and the framework since it was difficult to desorb His from the pores of NH_2_-MIL-101-Al. Such strong interactions together with the exchange of solvent molecules with His as mentioned above will increase the rigidity and decrease the non-radiative decay [48]; therefore, fluorescence enhancement was observed when His was added into the NH_2_-MIL-101-Al suspension. 

## 3. Materials and Methods

All chemicals were received from commercial sources and used directly without purification. The sample of NH_2_-MIL-101-Fe was fabricated according to the method reported previously [49] and the synthetic procedure of NH_2_-MIL-101-Al was followed according to the previously reported literature [50]. PXRD measurements were performed on a Bruker D8 X-ray diffractometer with a Cu-Kα radiation source (*λ* = 1.5418 Å) under 40 kV and 40 mA. FT-IR-ATR spectra within the range of 400–4000 cm^–1^ were recorded on an infrared spectrophotometer (Bruker Tensor II) with a diamond ATR module. Fluorescence and ^1^H NMR spectral data were obtained by using a Perkin Elmer LS-55 fluorescence spectrometer and a Bruker-DRX (500 MHz) NMR instrument, respectively. HRMS data were achieved on a Thermo Scientific Q Exactive electrospray mass spectrometer. 

The fluorescence properties of the as-synthesized samples of NH_2_-MIL-101-Fe and NH_2_-MIL-101-Al in the solid state and in aqueous suspension were investigated at room temperature. To investigate the sensing capability of NH_2_-MIL-101-Fe and NH_2_-MIL-101-Al toward AAs, powder samples of NH_2_-MIL-101-Fe and NH_2_-MIL-101-Al were dispersed in deionized water and aqueous solutions of different amino acids (0.1 M) with ultrasonic treatment to generate stable suspensions (0.5 mg mL^−1^), which can be used for four hours or longer after its preparation without any obvious fluorescence change. The fluorescence emission spectra of NH_2_-MIL-101-Fe and NH_2_-MIL-101-Al were recorded within the range of 375–690 and 360–660 nm upon excitation at 356 and 340 nm, respectively. To obtain reliable data, each test was repeated three times.

For the quantitative titration experiments, aqueous solution of Cys (0.02 M) was stepwise added to the aqueous suspension of NH_2_-MIL-101-Fe (0.5 mg mL^−1^, 3 mL), and aqueous solutions of Lys/Arg/His (0.1 M) were added stepwise to the suspension (water/ethanol = 1/1, *v*/*v*) of NH_2_-MIL-101-Al (0.5 mg mL^−1^, 3 mL). The fluorescence emission spectra were recorded after a fixed time interval after the addition of Cys/Lys/Arg/His. For the anti-interference experiments, the aqueous suspension of NH_2_-MIL-101-Fe (0.25 mg mL^−1^) and the suspension (water/ethanol = 1/1, *v*/*v*) of NH_2_-MIL-101-Al (0.25 mg mL^−1^) were added solutions of other AAs (0.1 M, 0.2 mL for NH_2_-MIL-101-Fe, 1 mL for NH2-MIL-101-Al) successively and aqueous solutions of Cys (0.02 M, 1 mL) and Lys/Arg/His (0.1 M, 1 mL) subsequently, and then the fluorescence emission spectra were recorded. Each test was repeated at least three times. 

## 4. Conclusions

In this work, we investigated the sensing capability of amino-functionalized MIL-101 with different metal centers, namely NH_2_-MIL-101-Fe and NH_2_-MIL-101-Al, toward natural amino acids. The results of sensing experiments demonstrated that NH_2_-MIL-101-Fe and NH_2_-MIL-101-Al could detect Cys and Lys/Arg/His via a fluorescence turn-on effect, respectively. The results of titration experiments show that the fluorescence enhancement has linear relationships with the concentrations of the analytes within a certain concentration range. Meanwhile, they also exhibit reasonable anti-interference except that Lys, Asp, His and Arg could affect the sensing capability of NH_2_-MIL-101-Fe toward Cys. Moreover, the sensing capacity of NH_2_-MIL-101-Fe for Cys and of NH_2_-MIL-101-Al for Lys/Arg could be ascribed to the structural collapse, while the detection mechanism of NH_2_-MIL-101-Al for His could be attributed to the adsorption of His into the pores of NH_2_-MIL-101-Al. Furthermore, the redox reaction between Cys and Fe^3+^ of NH_2_-MIL-101-Fe was responsible for the ultrahigh fluorescence enhancement. 

## Data Availability

Not applicable.

## Supplementary Materials

The following are available online: Figure S1: PXRD patterns of the as-synthesized NH_2_-MIL-101-Fe and NH_2_-MIL-101-Al and the simulated one; Figure S2: (a) Fluorescence emission spectra of NH_2_-MIL-101-Fe in the solid state and in aqueous suspension; (b) Fluorescence emission spectra of NH_2_-MIL-101-Al in the solid state and in aqueous suspension; Figure S3: (a) ^1^H NMR spectra in D_2_O of His, digested NH_2_-MIL-101-Al before and after immersion in the solution of His; (b) FT-IR spectra of His, NH_2_-MIL-101-Al before and after immersion in the aqueous solution of His; Figure S4: (a) ^1^H NMR spectra in D_2_O of Cys and digested NH_2_-MIL-101-Fe after immersion in the solution of Cys. (b) HRMS spectrum of digested NH_2_-MIL-101-Fe after immersion in aqueous solution of Cys; Figure S5: Fluorescence emission of NH_2_-BDC, NH_2_-MIL-101-Fe and NH_2_-MIL-101-Al in aqueous suspension.

## References

[1] Du R.R., Yang D.T., Jiang G.J., Song Y.R., Yin X.Q. (2020). An Approach for In Situ Rapid Detection of Deep-Sea Aromatic Ami-no Acids Using Laser-Induced Fluorescence. Sensors.

[2] Zhou Y., Yoon J. (2012). ChemInform Abstract: Recent Progress in Fluorescent and Colorimetric Chemosensors for Detection of Amino Acids. ChemInform.

[3] Pettiwala A.M., Singh P.K. (2018). Optical Sensors for Detection of Amino Acids. Curr. Med. Chem..

[4] Naqvi S.T.R., Rasheed T., Ashiq M.N., Haq M.N.U., Majeed S., Fatima B., Nawaz R., Hussain D., Shafi S. (2020). Fabrication of iron modified screen printed carbon electrode for sensing of amino acids. Polyhedron.

[5] Zhao G., Yang W., Li F., Deng Z., Hu Y. (2020). A turn-on fluorescent probe for real-time detection of endogenous cysteine in living cells. J. Lumin..

[6] Duan Z., Zhu Y., Yang Y., He Z., Liu J., Li P., Wang H., Tang B. (2019). Fluorescent Imaging for Cysteine Detection In Vivo with High Selectivity. ChemistryOpen.

[7] Lim S.-Y., Kim H.-J. (2011). Ratiometric detection of cysteine by a ferrocenyl Michael acceptor. Tetrahedron Lett..

[8] Xue S., Ding S., Zhai Q., Zhang H., Feng G. (2015). A readily available colorimetric and near-infrared fluorescent turn-on probe for rapid and selective detection of cysteine in living cells. Biosens. Bioelectron..

[9] Arendowski A., Ruman T. (2018). Lysine detection and quantification by laser desorption/ionization mass spectrometry on gold na-noparticle-enhanced target. Anal. Methods.

[10] Zhang M., Qiao J., Zhang S., Qi L. (2018). Copper nanoclusters as probes for turn-on fluorescence sensing of Llysine. Talanta.

[11] Cheng R., Ge C., Bu Y., Ou S., Xue Y., Dai L., Peng Y., Liu H., Huang H. (2017). A turn-on fluorescent lysine nanoprobe based on the use of the Alizarin Red aluminum(III) complex conjugated to graphene oxide, and its application to cellular imaging of lysine. Microchim. Acta.

[12] Liu H.J., Li M., Jiang L.Y., Shen F., Hu Y.F., Ren X.Q. (2017). Sensitive arginine sensing based on inner filter effect of Au nanopar-ticles on the fluorescence of CdTe quantum dots. Spectrochim. Acta Part A.

[13] Verma N., Singh A.K., Singh M. (2017). L-arginine biosensors: A comprehensive review. Biochem. Biophys. Rep..

[14] Cui R., Wan Y., Ji G., Liu Z. (2019). A highly selective and sensitive fluorescent sensor based on Tb^3+^-functionalized MOFs to determine arginine in urine: A potential application for the diagnosis of cystinuria. Analyst.

[15] He Y., Wang X., Zhu J.J., Zhong S.H., Song G.W. (2012). Ni^2+^-modified gold nanoclusters for fluorescence turn-on detection of his-tidine in biological fluids. Analyst.

[16] Xu C., Zhang S., Zang H., Yuan B., Fan R., Zhang N., Guo L., Niu Y., Zhang Y., Jin J. (2018). A Simple and Facile Electrochemi-cal Sensor for Sensitive Detection of Histidine Based on Three-Dimensional Porous Ni Foam. Int. J. Electrochem. Sci..

[17] Watanabe M., Suliman M.E., Qureshi A.R., Garcia-Lopez E., Barany P., Heimburger O., Stenvinkel P., Lindholm B. (2008). Con-sequences of low plasma histidine in chronic kidney disease patients: Associations with inflammation, oxidative stress, and mortality. Am. J. Clin. Nutr..

[18] Lu X.H., Wang W., Dong Q., Bao X.L., Lin X.F., Zhang W.X., Dong X.C., Zhao W.L. (2015). A multi-functional probe to discrimi-nate Lys, Arg, His, Cys, Hcy and GSH from common amino acids. Chem. Commun..

[19] Yu Q., Li Z., Cao Q., Qu S., Jia Q. (2020). Advances in luminescent metal-organic framework sensors based on post-synthetic modification. TrAC Trends Anal. Chem..

[20] Luo S., Zeng Z., Zeng G., Liu Z., Xiao R., Chen M., Tang L., Tang W., Lai C., Cheng M. (2019). Metal Organic Frameworks as Robust Host of Palladium Nanoparticles in Heterogeneous Catalysis: Synthesis, Application, and Prospect. ACS Appl. Mater. Interfaces.

[21] Zhang Y., Yang L., Wang L., Duttwyler S., Xing H. (2019). A Microporous Metal-Organic Framework Supramolecularly Assembled from a Cu^II^ Dodecaborate Cluster Complex for Selective Gas Separation. Angew. Chem. Int. Ed..

[22] Li P., Shen Y., Wang D., Chen Y., Zhao Y. (2019). Selective Adsorption-Based Separation of Flue Gas and Natural Gas in Zirconium Metal-Organic Frameworks Nanocrystals. Molecules.

[23] Shu Y., Ye Q., Dai T., Xu Q., Hu X. (2021). Encapsulation of Luminescent Guests to Construct Luminescent Metal–Organic Frameworks for Chemical Sensing. ACS Sens..

[24] Liu X.B., Liang T.T., Zhang R.T., Ding Q., Wu S.Q., Li C.H., Lin Y., Ye Y., Zhong Z.R., Zhou M.L. (2021). Iron-Based Met-al-Organic Frameworks in Drug Delivery and Biomedicine. ACS Appl. Mater. Interfaces.

[25] Butler K.S., Pearce C.J., Nail E.A., Vincent G.A., Gallis D.F.S. (2020). Antibody Targeted Metal–Organic Frameworks for Bioimaging Applications. ACS Appl. Mater. Interfaces.

[26] Zhang Y., Yuan S., Day G., Wang X., Yang X., Zhou H.-C. (2018). Luminescent sensors based on metal-organic frameworks. Coord. Chem. Rev..

[27] Yi F.-Y., Chen D., Wu M.-K., Han L., Jiang H.-L. (2016). Chemical Sensors Based on Metal-Organic Frameworks. ChemPlusChem.

[28] Chen B., Xiang S., Qian G. (2010). Metal−Organic Frameworks with Functional Pores for Recognition of Small Molecules. Acc. Chem. Res..

[29] Dong J., Zhao D., Lu Y., Sun W.Y. (2019). Photoluminescent metal-organic frameworks and their application for sensing biomole-cules. J. Mater. Chem. A.

[30] Nagarkar S., Joarder B., Chaudhari A.K., Mukherjee S., Ghosh S.K. (2013). Highly Selective Detection of Nitro Explosives by a Luminescent Metal-Organic Framework. Angew. Chem. Int. Ed..

[31] Chen B., Wang L., Zapata F., Qian G., Lobkovsky E.B. (2008). A Luminescent Microporous Metal−Organic Framework for the Recognition and Sensing of Anions. J. Am. Chem. Soc..

[32] Gassensmith J.J., Kim J.Y., Holcroft J.M., Farha O.K., Stoddart J.F., Hupp J.T., Jeong N.C. (2014). A Metal-Organic Frame-work-Based Material for Electrochemical Sensing of Carbon Dioxide. J. Am. Chem. Soc..

[33] Yu Y., Zhang X.-M., Ma J.-P., Liu Q.-K., Wang P., Dong Y.-B. (2014). Cu(i)-MOF: Naked-eye colorimetric sensor for humidity and formaldehyde in single-crystal-to-single-crystal fashion. Chem. Commun..

[34] Cui Y., Xu H., Yue Y., Guo Z., Yu J., Chen Z., Gao J., Yang Y., Qian G., Chen B. (2012). A Luminescent Mixed-Lanthanide Metal–Organic Framework Thermometer. J. Am. Chem. Soc..

[35] Harbuzaru B.V., Corma A., Rey F., Jorda J.L., Ananias D., Carlos L., Rocha J. (2009). A Miniaturized Linear pH Sensor Based on a Highly Photoluminescent Self-Assembled Europium(III) Metal-Organic Framework. Angew. Chem. Int. Ed..

[36] Kanan S.M., Malkawi A. (2020). Recent Advances in Nanocomposite Luminescent Metal-Organic Framework Sensors for Detecting Metal Ions. Comments Inorg. Chem..

[37] Li B.Z., Suo T.Y., Xie S.Y., Xia A.Q., Ma Y.J., Huang H., Zhang X., Hu Q. (2021). Rational design, synthesis, and applications of carbon dots@metal-organic frameworks (CD@MOF) based sensors. TrAC Trends Anal. Chem..

[38] Dong J., Zhang X.-D., Xie X.-F., Guo F., Sun W.-Y. (2020). Amino group dependent sensing properties of metal–organic frameworks: Selective turn-on fluorescence detection of lysine and arginine. RSC Adv..

[39] Kandiah M., Nilsen M.H., Usseglio S., Jakobsen S., Olsbye U., Tilset M., Larabi C., Quadrelli E.A., Bonino F., Lillerud K.P. (2010). Synthesis and Stability of Tagged UiO-66 Zr-MOFs. Chem. Mater..

[40] Park K.S., Ni Z., Côté A.P., Choi J.Y., Huang R., Uribe-Romo F., Chae H.K., O’Keeffe M., Yaghi O.M. (2006). Exceptional chemical and thermal stability of zeolitic imidazolate frameworks. Proc. Natl. Acad. Sci. USA.

[41] Serre C., Millange F., Thouvenot C., Nogues M., Marsolier G., Louer D., Ferey G. (2002). Very Large Breathing Effect in the First Nanoporous Chromium(III)-Based Solids: MIL-53 or Cr^III^(OH)·{O_2_C−C_6_H_4_−CO_2_}·{HO_2_C−C_6_H_4_−CO_2_H}_x_·H_2_O_y_. J. Am. Chem. Soc..

[42] Rosi N.L., Eckert J., Eddaoudi M., Vodak D.T., Kim J., O’Keeffe M., Yaghi O.M. (2003). Hydrogen storage in microporous met-al-organic frameworks. Science.

[43] Li H., Eddaoudi M., O’Keeffe M., Yaghi O.M. (1999). Design and synthesis of an exceptionally stable and highly porous met-al-organic framework. Nature.

[44] Hong D.-Y., Hwang Y.K., Serre C., Férey G., Chang J.-S. (2009). Porous Chromium Terephthalate MIL-101 with Coordinatively Unsaturated Sites: Surface Functionalization, Encapsulation, Sorption and Catalysis. Adv. Funct. Mater..

[45] Zhao D., Liu X.-H., Zhao Y., Wang P., Liu Y., Azam M., Al-Resayes S.I., Sun W.-Y. (2017). Luminescent Cd(II)–organic frameworks with chelating NH_2_ sites for selective detection of Fe(III) and antibiotics. J. Mater. Chem. A.

[46] Höller R.P.M., Jahn I.J., Cialla-May D., Chanana M., Popp J., Fery A., Kuttner C. (2020). Biomacromolecular-Assembled Nanoclusters: Key Aspects for Robust Colloidal SERS Sensing. ACS Appl. Mater. Interfaces.

[47] Fazio B., D’Andrea C., Foti A., Messina E., Irrera A., Donato M.G., Villari V., Micali N., Marago O.M., Gucciardi P.G. (2016). SERS detection of Biomolecules at Physiological pH via aggregation of Gold Nanorods mediated by Optical Forces and Plasmonic Heating. Sci. Rep..

[48] Kitagawa S., Kitaura R., Noro S.-I. (2004). Functional Porous Coordination Polymers. Angew. Chem. Int. Ed..

[49] Dao X.-Y., Guo J., Wei Y.-P., Guo F., Liu Y., Sun W.-Y. (2019). Solvent-Free Photoreduction of CO_2_ to CO Catalyzed by Fe-MOFs with Superior Selectivity. Inorg. Chem..

[50] Crespo P.S., Ramos-Fernandez E.V., Gascon J., Kapteijn F. (2011). Synthesis and Characterization of an Amino Functionalized MIL-101(Al): Separation and Catalytic Properties. Chem. Mater..

