# Supplementary material for: Sensing Properties of NH2-MIL-101 Series for Specific Amino Acids via Turn-On Fluorescence
Source: Molecules. 2021 Sep 2;26(17):5336. doi: 10.3390/molecules26175336 (PMC8434598; doi:10.3390/molecules26175336)
Supplement: Supplementary file 1 [file molecules-26-05336-s001.zip › molecules-1366079-supplementary.pdf]

Supplementary Material

Sensing Properties of NH_2-MIL-101 Series for Specific Amino Acid via Turn-On Fluorescence

Jing Dong [1,3], Xiao-Yao Dao [1], Xiao-Yu Zhang [1], Xiu-Du Zhang [2,*] and Wei-Yin Sun [1,*]

[1] Coordination Chemistry Institute, State Key Laboratory of Coordination Chemistry, School of Chemistry and Chemical Engineering, Nanjing National Laboratory of Microstructures, Collaborative Innovation Center of Advanced Microstructures, Nanjing University, Nanjing 210023, China; jdong@njtech.edu.cn (J.D.); DG1724013@smail.nju.edu.cn (X.-Y.D); DG20240143@smail.nju.edu.cn (X.-Y.Z.)

[2] College of Chemistry and Materials Science, Key Laboratory of Functional Molecular Solids Ministry of Education, Anhui Laboratory of Molecule-Based Materials, Anhui Key Laboratory of Functional Molecular Solids, Anhui Normal University, Wuhu 241002, China

[3] Nanjing Tech University, Nanjing 211816, China

* Correspondence: xiuduzhang@ahnu.edu.cn (X.-D.Z.); sunwy@nju.edu.cn (W.-Y.S.)

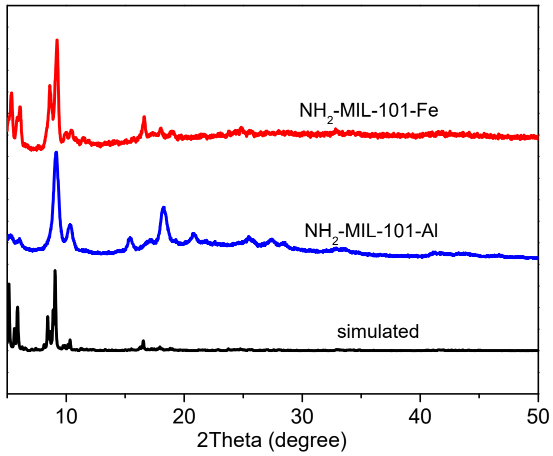

Figure S1. PXRD patterns of the as-synthesized NH_2-MIL-101-Fe and NH_2-MIL-101-Al and the simulated one.

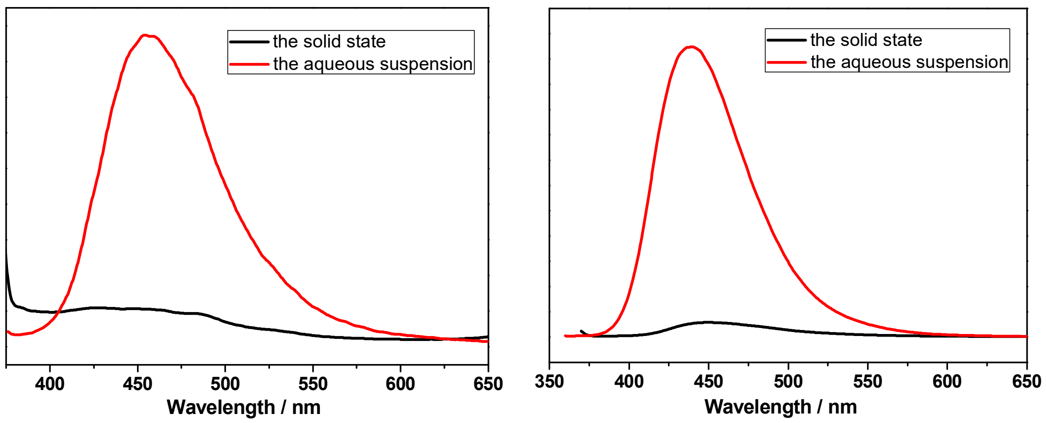

(a) (b)

Figure S2. (a) Fluorescence emission spectra of NH_2-MIL-101-Fe in the solid state and aqueous suspension. (b) Fluorescence emission spectra of NH_2-MIL-101-Al in the solid state and aqueous suspension.

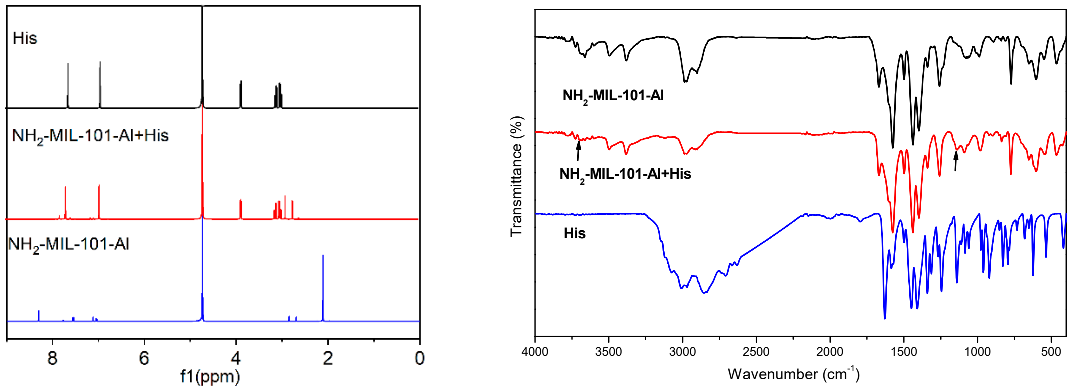

(a) (b)

Figure S3. (a) ^{1}H-NMR spectra in D_2O of His, digested NH_2-MIL-101-Al before and after the immersion in the solution of His. (b) FT-IR spectra of His, NH_2-MIL-101-Al before and after the immersion in the aqueous solution of His.

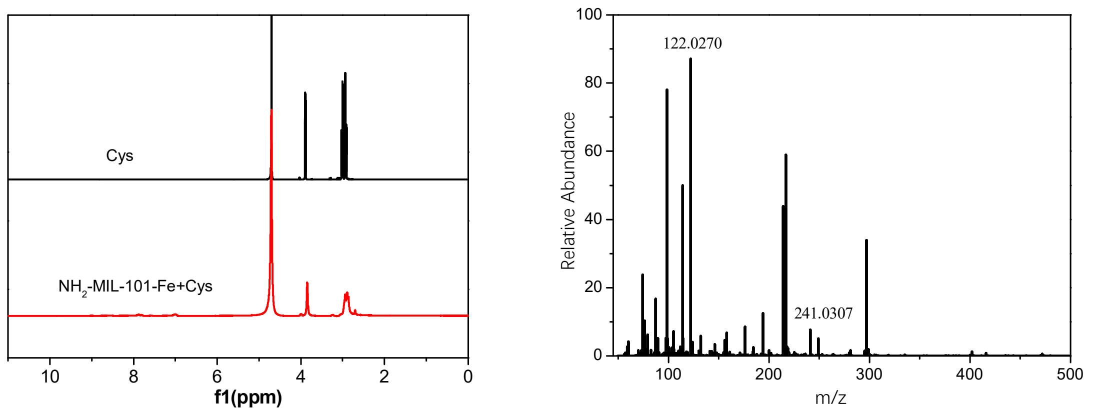

(a) (b)

Figure S4. (a) ^{1}H-NMR spectra in D_2O of Cys and digested NH_2-MIL-101-Fe after the immersion in the solution of Cys. (b) HRMS spectrum of digested NH_2-MIL-101-Fe after the immersion in aqueous solution of Cys.
